# Combining Ultrafiltration and Nanofiltration to Obtain a Concentrated Extract of Purified Polyphenols from Wet Olive Pomace

**DOI:** 10.3390/membranes13020119

**Published:** 2023-01-17

**Authors:** Carmen M. Sánchez-Arévalo, Ane Pérez García-Serrano, María Cinta Vincent-Vela, Silvia Álvarez-Blanco

**Affiliations:** 1 Research Institute for Industrial, Radiophysical and Environmental Safety (ISIRYM), Universitat Politècnica de València, Camino de Vera, s/n, 46022 Valencia, Spain; 2 Department of Chemical and Nuclear Engineering, Universitat Politècnica de València, Camino de Vera s/n, 46022 Valencia, Spain

**Keywords:** ultrafiltration, nanofiltration, phenolic compounds, wet olive pomace, integrated process, rejection

## Abstract

Despite the environmental concerns raised every year by the generation of high volumes of wet olive pomace, it contains valuable phenolic compounds that are essential for the valorization of this by-product. In this work, an integrated process to recover phenolic compounds from wet olive pomace is proposed. It consists of ultrasound-assisted solid-liquid extraction, followed by ultrafiltration and nanofiltration. Several commercial membranes were studied at different operational conditions. The ultrafiltration stage allowed the purification of biophenols, which were obtained in the permeate stream. Regarding organic matter, satisfactory rejection values were obtained with both commercial UH030 and UP005 membranes (Microdyn Nadir), but the latter provided more efficient purification and higher values of permeate flux, above 18 L·h^−1^·m^−2^ at 2.5 bar and 1.5 m·s^−1^. Later, this permeate stream was concentrated by means of a nanofiltration process, obtaining polyphenol rejection values that surpassed 85% with the commercial NF270 membrane (DuPont), then achieving the concentration of the previously purified polyphenols.

## 1. Introduction

Annual production of virgin olive oil ends with the generation of this valuable product and, inevitably, tons of residues derived from the processing of olives [1]. As a result of the application of the two-phase methodology in an olive mill, wet olive pomace is produced. It is a semi-solid by-product, containing the remains of olive pulp, stone, skin and vegetation water. It comprises a concerning residue due to its phytotoxic character and high organic load [2]. Therefore, its treatment and detoxification preceding its disposal is of high importance.

Additionally, the principles of circular economy that have gained relevance in recent years motivate the valorization of this by-product in order to incorporate it back into the consumption chain [3]. In the context of the olive mill, wet olive pomace can be employed as a source of high-added-value compounds due to its high content of phenolic compounds. These molecules are able to reduce oxidant chemical species (reactive oxygen species, for instance), then preventing the oxidation of other compounds, such as essential biomolecules. Apart from their powerful antioxidant capacity, several authors have described their antiproliferative, anti-inflammatory and antibiotic effects, among others [4,5,6]. This repertoire of significant properties determines the application of polyphenols in the pharmaceutical, cosmetic and nutraceutical fields [7].

The content of phenolic compounds can be retrieved from wet olive pomace by means of solid–liquid extraction. Several techniques have been investigated with this objective, such as agitation, maceration, pressurized liquid extraction and ultrasound-assisted extraction [8,9,10,11]. In most cases, the extraction is efficient, but the obtained polyphenols are either not highly pure or very diluted. The combination of both scenarios is possible too. For these reasons, the implementation of membrane technology is an excellent strategy. It allows the possibility of working in mild operating conditions, with low energy consumption, excellent separation efficiency and control over this separation efficiency [12,13]. Furthermore, this technology permits working continuously and at smaller facilities, being environment-friendly and based on nonharmful materials [14]. The efficiency of membrane processes to treat and valorize agri-food residues has already been demonstrated. Ultrafiltration has been effectively applied to recover phenolic compounds from Eucalyptus bark [15], olive oil washing wastewater [16] or grape pomace [17]. Furthermore, a sequential process can be designed, combining ultrafiltration and nanofiltration [18], or microfiltration and nanofiltration [19]. These integrated processes logically require operating in concentration mode. Despite what it may seem, this is not trivial, because the constant increment of feed concentration greatly affects membrane fouling and, consequently, the permeate flux. Studies in recirculation mode are enormously useful during the membrane selection stage, as the concentration in the feed tank is kept constant, but assessing membrane performance in concentration mode during extended periods is mandatory if the industrial application is to be considered. Some scientific contributions applying membrane technology and working in concentration mode to recover polyphenols from wet olive pomace are summarized in Table 1.

This premise regarding the concentration mode was considered in this work. In this study, an integrated process consisting of ultrafiltration and subsequent nanofiltration of an aqueous extract of wet olive pomace was investigated. A double aim was pursued. On one side, the reduction of the environmental impact of a major residue from an extended industry, as it is the olive oil sector; and, on the other side, the recovery of valuable compounds such as olive-derived polyphenols.

To that end, polymeric commercial membranes were employed to treat aqueous extracts of wet olive pomace through ultrafiltration and subsequent nanofiltration. The extracts of wet olive pomace are brown liquids, rich in sugars, phenolic compounds, triterpenes, organic acids and free fatty acids [8,23]. Considering this composition, an ultrafiltration process can be implemented to remove the undesired compounds and purify the polyphenols of interest. The obtained stream can be later concentrated by means of a nanofiltration process, obtaining a profitable product out of a challenging residue.

## 2. Materials and Methods

### 2.1. Reagents and Raw Material

Wet olive pomace was obtained from the two-phase olive mill San Isidro Cooperative (Segorbe, Castellón, Spain) during the olive campaign of 2021/2022. After their collection, samples were refrigerated at 5 °C to preserve them. The Folin–Ciocalteu reagent was provided by MP Biomedicals (Ilkirch, France). To prepare the mobile phases for chromatography, acetonitrile was purchased from Honeywell (Charlotte, NC, USA), and osmotized water was obtained from a Direct-Q^®^, 3UV system (Burlington, MA, Merck Millipore, USA). Pure standards of tyrosol, hydroxytyrosol and oleuropein were purchased from Bionova Científica (Madrid, Spain). Sigma-Aldrich (Saint Louis, MI, USA) provided the standards for caffeic acid, luteolin and *p*-coumaric acid.

### 2.2. Extraction of Polyphenols

The phenolic content from the wet olive pomace was extracted according to a previously optimized methodology [8]. Briefly, 600 g of wet olive pomace were subjected to ultrasound-assisted extraction (UAE), employing osmotized water as the extractant. The UAE was performed at 40 °C for 45 min. Afterwards, the sample was centrifuged at 17,200× *g* RCF for 6 min, and the resulting extract was vacuum filtered with a 60 µm filter (Fanoia, Barcelona, Spain) and subsequently treated by membrane technology.

### 2.3. Membrane Processes

**Figure 1:**
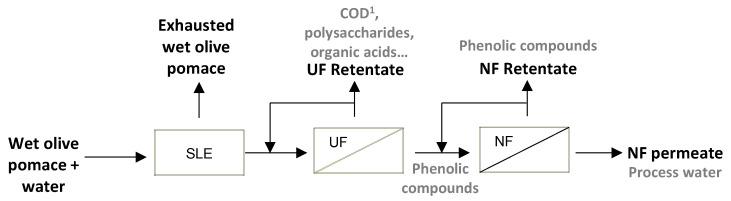
Schematic diagram of the recovery of phenolic compounds from wet olive pomace by the proposed integrated process consisting of solid–liquid extraction (SLE), ultrafiltration (UF) and nanofiltration (NF). ^1^ COD: chemical oxygen demand.

A simplified scheme of the proposed procedure to purify phenolic compounds from wet olive pomace can be found in Figure 1.

#### 2.3.1. Ultrafiltration Process

The aqueous extract of wet olive pomace was ultrafiltered in an ultrafiltration cross-flow plant (Orelis Environnement, Salindres, France). A Rayflow membrane module (Orelis Environnement, Salindres, France) contained two ultrafiltration membranes (Microdyn-Nadir, Wiesbaden, Germany) working in series. Each membrane was tested in a different run, in order to control the variation of the permeate flux with the volume reduction factor (VRF). The information about the tested membranes is given in Table 2. Microscopic characterization of the considered ultrafiltration membranes can be found in [24,25,26].

Prior to their utilization, the membranes were immersed in osmotized water overnight in order to hydrate them and remove any conservative remnants. Then, they were compacted with osmotized water at cross-flow velocity of 1.5 m·s^−^^1^ and transmembrane pressure (***TMP***) of 3 bar until stable permeate flux was observed. The hydraulic permeability ($L_{w}$) of the membranes was also determined in the range of 1–2.5 bar at 1 m·s^−^^1^ according to the following equation:

(1) $$L_{w}=\frac{J_{w}}{TMP}$$

where $J_{w}$ represents water permeate flux.

Afterwards, the extract was processed at 2.5 bar and 1.5 m·s^−^^1^. These conditions were selected according to previous experience from our research group [16,30,31] and preliminary experiments (manuscript in preparation). Each membrane had an area of 129 cm^2^. This process was carried out in concentration mode in order to collect the ultrafiltration permeate to be treated in a subsequent nanofiltration stage. The permeate was collected until a VRF of at least 2 was achieved. In consequence, these experiments were continued for several weeks. At the end of each working day, the feed solution was removed from the ultrafiltration plant, and the membrane was rinsed for 15 min with water to reduce the accumulation of residues in the plant and avoid the development of organic fouling during the night. The next working day, the procedure was resumed. When necessary, chemical cleaning had to be done during the ultrafiltration process, as detailed in Section 2.3.3.

#### 2.3.2. Nanofiltration Process

The ultrafiltration permeate was then treated by nanofiltration in an HP4750 bench-top cell (Sterlitech, Auburn, Washington, DC, USA), with a membrane area of 14.6 cm^2^. The NF270 membrane (DuPont-Filmtec, Wilmington, DE, USA) was employed. It was previously immersed in osmotized water (for at least 12 h) and compacted at 9.5 bar. The hydraulic permeability of the membrane was tested in the range of 5–9 bar. Afterwards, several TMPs (5, 7 and 9 bar) were tested to treat the ultrafiltration permeate, until a VRF of 2.5 was achieved. The feed solution was constantly stirred at 500 rpm.

#### 2.3.3. Membrane Cleaning

To clean the ultrafiltration membranes, the first rinsing with tap water was followed by cleaning with a solution of P3 Ultrasil (Ecolab, Saint Paul, MN, USA) at 1% (*v*/*v*) and 35 °C. The chemical cleaning was maintained for 1 h at 1.5 bar and 1.5 m·s^−1^. Afterwards, the membranes were again rinsed with tap water until the detergent was totally withdrawn from the module. This was monitored by measuring the pH of the permeate and comparing it with the pH of tap water. The membranes were cleaned after each filtration experiment with the wet olive pomace. Additionally, a cleaning step had to be introduced during the long-term ultrafiltration operation with the UH030 membrane.

The cleaning for the nanofiltration step was dependent on the feed that was treated. Thus, the membrane was cleaned by a simple rinse with tap water, without the application of any temperature or pressure, after the treatment of the permeate from the UP005 membrane. When the permeate from the UH030 membrane was treated by nanofiltration, a solution of P3 Ultrasil at 1% (*v*/*v*) and 35 °C was employed to clean the membrane. This cleaning consisted of filtration of 200 mL of the chemical solution at 2.5 bar, followed by rinsing with tap water.

### 2.4. Characterization of the Streams

Samples from the feed solution, retentate, global permeate (corresponding to the global product obtained during the whole process) and instantaneous permeate (corresponding to the permeate obtained during the final minutes of the process) were characterized. All samples were analyzed at least in duplicates. Rejection of the solutes (R) was calculated according to the following equation:

(2) $$R\;=\;\left(1-\frac{C_{p}}{C_{r}}\right)\cdot 100$$

where $C_{p}$ is the concentration in the instantaneous permeate and $C_{r}$ is the concentration in the retentate stream.

The chemical oxygen demand (COD) of the samples was analyzed by means of the commercial Spectroquant^®^ COD Test Cells (Merck, Darmstadt, Germany). The total solids content was assessed through evaporation of a determined volume and consecutive weighing of the dry sample. pH (Crison, Barcelona, Spain) of the samples was also monitored. The Folin–Ciocalteu methodology was employed to determine the total phenolic content of the analyzed streams [32]. A pure standard of tyrosol was used to perform the external calibration of the analysis, then expressing the results as mg tyrosol/L. Additionally, the phenolic profile of the streams derived from the UP005 membrane was assessed through liquid chromatography coupled to mass spectrometry (LC-MS). To that end, a previously optimized methodology was applied [8]. Shortly, the samples were filtrated using 0.2 μm filters (ThermoFisher, Waltham, MA, USA), and the analytes were separated employing a 1260 Infinity II LC system equipped with a Zorbax Extend C18 column (4.6 × 100 mm, 1.8 μm) (Agilent Technologies, Santa Clara, CA, USA). Acidified acetonitrile and acidified water (containing 0.5% (*v*/*v*) of acetic acid) were employed to perform the gradient of the mobile phases. This instrument was coupled to a 6546 quadrupole-time-of-flight (qToF) mass analyzer (Agilent Technologies, Santa Clara, CA, USA), working in negative polarity. Electrospray (ESI) was employed as the interface. Samples were injected at least in duplicates and quantified by external calibration.

## 3. Results and Discussion

### 3.1. Aqueous Extract of Wet Olive Pomace

The characterization of the aqueous extract of wet olive pomace, employed as a feed solution for the process, is shown in Table 3. The study and optimization of the extraction stage were previously published [8]. As reflected, the COD and total solids content of the extract are considerable, making necessary the application of an ultrafiltration process to purify the extracted phenolic content. A relevant concentration of phenolic compounds is present in the wet olive pomace, enabling this residue as a source of high-added-value molecules.

### 3.2. Performance of Ultrafiltration

#### 3.2.1. Permeate Flux

**Figure 2:**
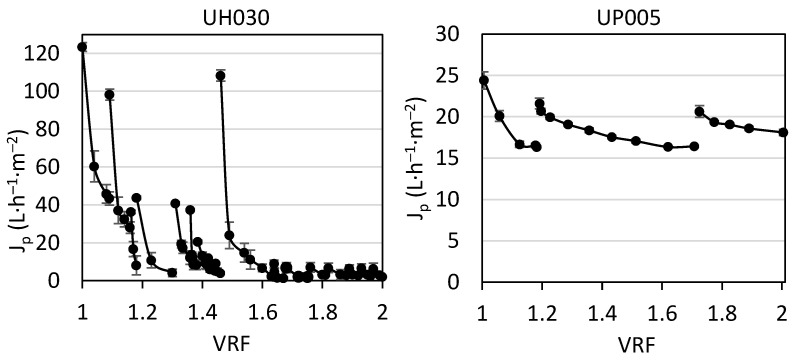
Evolution of permeate flux with the volume reduction factor (VRF) for the UH030 membrane (**left**) and the UP005 membrane (**right**) when the extract was treated.

After the compaction, the hydraulic permeability of each membrane was investigated, obtaining 85.7 ± 0.9 L·h^−^^1^·m^−^^2^·bar^−^^1^ for the UH030 membrane and 15 ± 1 L·h^−^^1^·m^−^^2^·bar^−^^1^ for the UP005 membrane. The permeate flux obtained with the UH030 and UP005 membranes when the extract was treated can be found in Figure 2.

As can be seen in Figure 2, seventeen ultrafiltration stages were needed to achieve a VRF of 2 with the UH030 membrane. In the first stage, a sharp decline of the permeate flux occurred due to severe membrane fouling [33,34]. At the beginning of the next stage, the permeate flux started at higher values with respect to the end of the first stage, and a sharp flux decline was again exhibited. This was observed because the membrane rinsing (performed at the end of each working day, as detailed in Section 2.3.1) was able to remove the incipient cake layer that was formed from the beginning of the process. The restoring of the permeate flux contributed to maintaining the efficiency of the procedure as the VRF increased, whereby the aqueous rinsing was considered adequate and it was implemented at the end of every working day. From the third stage, the initial value of the permeate flux decreased. Even though a similar curve for the flux decline was obtained every working day, the high initial values observed during the first and second stages were not obtained anymore. This was an indication of the thickening and tightening of the cake layer, which occurred from the VRF 1.15 until the VRF 1.5. The higher concentration of the feed solution (at higher VRF values) prompted a more severe concentration polarization that maximized the effect of the cake layer and membrane fouling [35,36]. As can be seen in Figure 2, the initial flux of the ninth ultrafiltration stage was more than four times lower than the initial permeate flux at VRF 1. In consequence, a chemical cleaning with P3 Ultrasil 115 1% (*v*/*v*) at 35 °C was performed (Section 2.3.3). Two cleaning cycles were needed to recover the hydraulic permeability of the UH030 membrane due to the severe fouling, accumulated during the extended operation time of the ultrafiltration. The permeability of the cleaned UH030 membrane was 85.2 L·h^−^^1^·m^2^·bar^−^^1^. At the beginning of the tenth stage, the high initial permeate flux was restored; however, the flux decrease was again fast, as in the first and second working cycles, when the membrane was not fouled yet. In this case, membrane rinsing did not lead to satisfactory values of permeate flux, even at the beginning of the next stages, because a strong cake layer was formed from the VRF of 1.6 henceforth.

The right panel of Figure 2 shows the permeate flux obtained with the UP005 membrane, which was less affected by fouling. If the flux of both membranes is compared, at the beginning of the process (VRF near 1), the permeate flux was much higher for the UH030 membrane. Therefore, during the first working stage, the UH030 membrane was considered to be more productive. This was expected because at this low concentration level, the concentration polarization and fouling were still low. Then, the membrane with the highest MWCO (see Table 2) exhibited the highest permeate flux. However, as the ultrafiltration progressed, severe fouling was suffered by the UH030 membrane, in contrast with the UP005 membrane. This discrepancy can be attributed to several different characteristics of the membranes. First, the UH030 membrane has a larger pore size, more likely to suffer from pore blocking. This phenomenon was quite plausible here, considering the complexity of agri-food samples, such as the aqueous extract of wet olive pomace, which contains numerous organic molecules from different chemical families and a wide range of molecular weights. Other authors have also described the pore blocking of membranes with higher MWCO in comparison with tighter ultrafiltration membranes. Lujan-Facundo et al. found that bovine serum albumin blocked the pores of the UH030 membrane, leading to significant fouling [30]. Similarly, Corbatón-Báguena et al. [37] and Qu et al. [38] described that solutes with a similar size to the membrane pores can penetrate inside them and reduce the flux, obtaining better results with membranes of smaller pore size, whose pores cannot be blocked by larger molecules. Furthermore, it has been described that membranes with higher permeability (such as the UH030 membrane in this case) are more likely to suffer from gel layer formation [16], which determines a reduction in the permeate flux and greatly hinders membrane cleaning, as was commented before. Another parameter that influenced the behavior of these membranes regarding the permeate flux is the polarity of the membrane surface. In this regard, the contact angle of the UH030 and UP005 membranes is reported in Table 2. Both membranes are made of polyethersulfone. The contact angle of both membranes is similar and lower than 90°, which indicates a hydrophilic character. According to the manufacturer, the UH030 membrane has been modified to increase its hydrophilicity. Therefore, lower values of contact angle for this membrane could be expected. However, the roughness of the UH030 membrane is 12.12 ± 3.16 nm, whereas the UP005 membrane presents a roughness of 1.59 ± 0.20 nm [28]. According to several authors [28,39,40], rougher membranes can display higher contact angles than more hydrophobic membranes with lower roughness. Furthermore, several authors have demonstrated that membranes with higher roughness suffer from more severe fouling [41,42], as was the case for the UH030 membrane in this work. The difference in hydrophilicity between the UP005 and UH030 membranes can be essential when the feed solution includes foulants such as phenolic compounds. Phenolic compounds have been demonstrated to contribute highly to irreversible fouling of ultrafiltration membranes due to an adsorption process [43]. The affinity between those compounds and the membrane surface can determine to a great extent their adsorption [44] and, consequently, membrane fouling. Cifuentes-Cabezas et al. determined that the adsorption of phenolic compounds on the surface of the UP005 membranes was 0.349 ± 0.014 mg·m^−^^2^. These authors found higher adsorption of phenolic compounds (0.465 ± 0.037 mg·m^−^^2^) on the active layer of the UH050 membrane, whose material is the same as that of the UH030 membrane [16]. As described by Cassano et al. [45] the higher polarity of the hydrophilic polyethersulfone (as in the UH030 membrane) determines a stronger interaction with the polyphenols from wet olive pomace, leading to stronger fouling.

The less hydrophilic active layer and the lower MWCO of the UP005 membrane led to reduced fouling and therefore, flux decline was much lower for this membrane. In consequence, fewer working stages were needed to achieve a VRF of 2. This is in agreement with the work of Cifuentes-Cabezas et al., who also observed that the UP005 did not suffer from severe fouling during ultrafiltration of olive oil washing wastewater [16]. As can be seen in Figure 2, a simple rinsing with osmotized water at the end of every working day was effective enough to remove the fouling layer from the UP005 membrane and restore a high permeate flux, which was maintained until the end of the ultrafiltration procedure, despite the progressive concentration of the feed solution.

**Figure 3:**
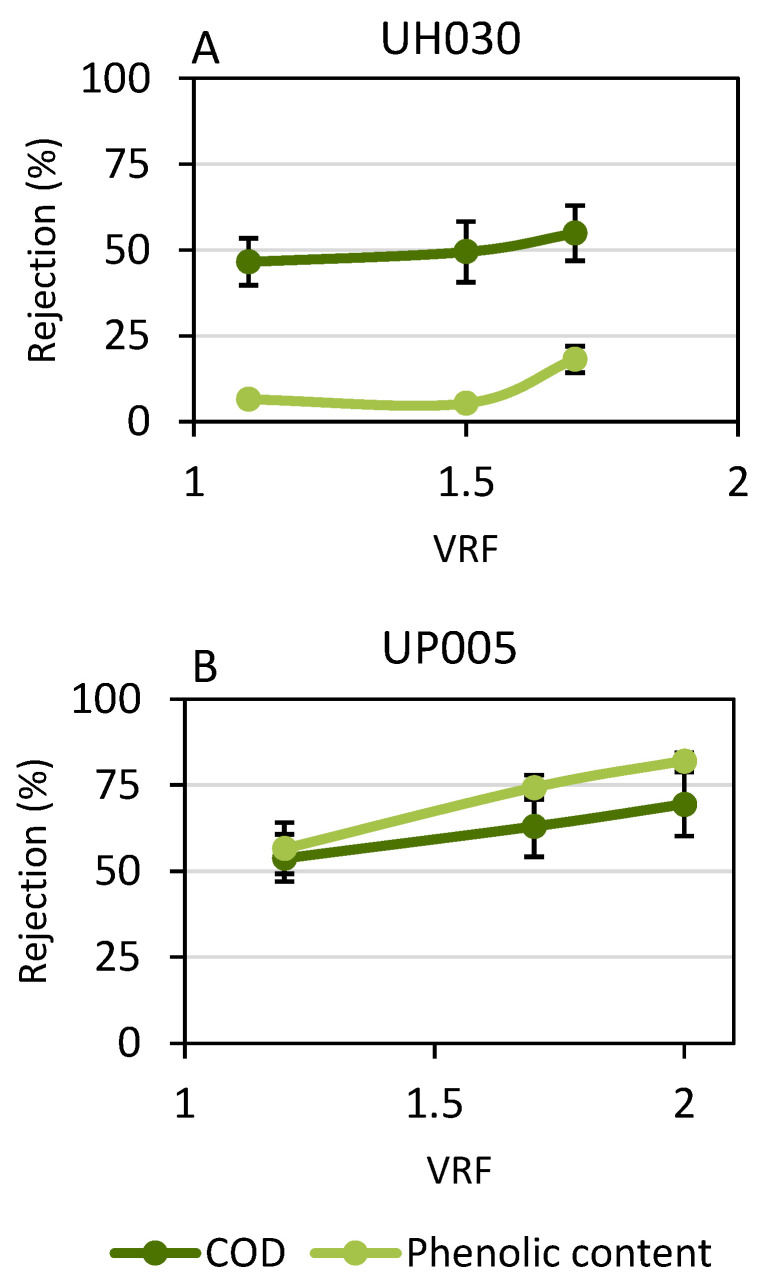
Rejection values obtained at the different volume reduction factors (VRFs) for the UH030 membrane (**A**) and the UP005 membrane (**B**).

From the third stage until the end of the global process (Figure 2), very similar values of permeate flux were exhibited by the UH030 membrane at the end of every stage. Each decline curve ended in low values of permeate flux, in the range of 1.3–5.4 L·h^−^^1^·m^2^, irrespective of the VRF. The concentration factor and applied rinsing or cleaning only influenced the capacity of removing the existent cake layer (in order to address the following stage), but the final value of the permeate flux was inevitably low. This, along with the results presented in Figure 3, motivated the selection of the UP005 membrane for the integrated process.

#### 3.2.2. Rejection Values

The rejection values obtained with both ultrafiltration membranes are presented in Figure 3.

The effect of the size exclusion phenomenon can be observed in the graphs presented in Figure 3. The UH030 membrane (Figure 3A) barely retained the phenolic content from the wet olive pomace because of its larger pore size. On the contrary, the obtained values for the rejection of the COD were higher, reaching 55 ± 8%. The increment in the rejection of the COD with the VRF occurred due to the strong fouling of the membrane that was previously commented on during the discussion of Figure 2. The high concentration of solutes near the membrane surface prompted the formation of an additional layer which contributed to the retention of large solids, such as proteins or polysaccharides.

**Figure 4:**
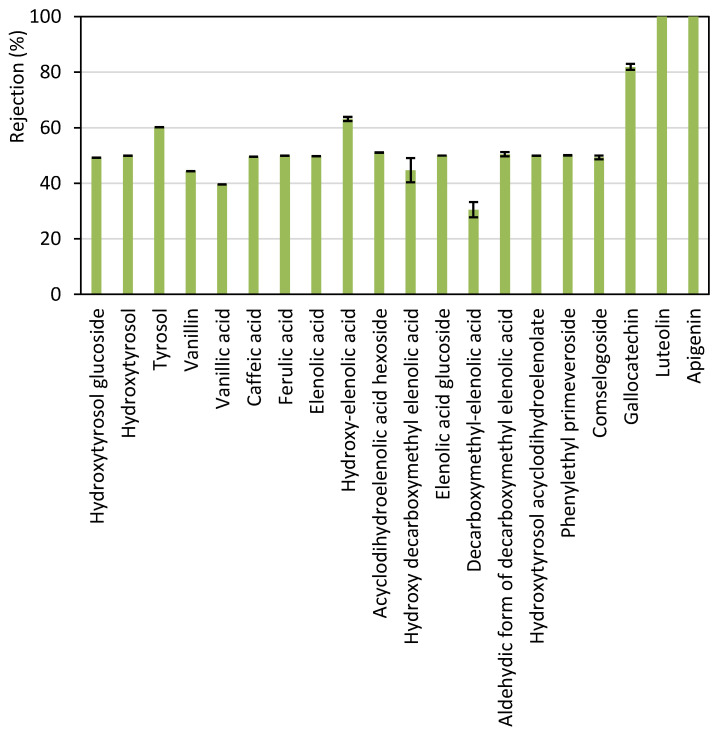
Rejection of each phenolic compound detected in the aqueous extract of wet olive pomace, obtained with the UP005 membrane at a volume reduction factor of 2. The operational conditions were 1.5 m·s^−1^ and 2.5 bar.

In the case of the UP005 membrane, a gradual increment could also be observed for COD rejection as an effect of membrane fouling. For this membrane, COD rejection reached 70 ± 2%, which was considered to be sufficient. The rejection of phenolic compounds obtained with the UP005 membrane was unexpectedly high, considering the results reported in the literature dealing with olive-derived wastewater [16,33]. Therefore, a more detailed analysis of the phenolic content of the streams derived from the UP005 was performed. These results are shown in Figure 4.

The permeate stream obtained with the UP005 membrane was analyzed by LC-ESI-qTOF-MS in order to identify the individual compounds present in the sample and the assessed rejection for each of them. This characterization provided more detailed information than the Folin–Ciocalteu methodology, which only rendered a global rejection value. Four chemical families of phenolic compounds were found in the samples. These were simple phenols (including tyrosol, hydroxytyrosol and hydroxytyrosol glucoside), phenolic acids and aldehydes (including vanillin, vanillic acid, caffeic acid and ferulic acid), secoiridoids (elenolic acid, hydroxy-elenolic acid, acyclodihydroelenolic acid, hydroxy-decarboxymethyl elenolic acid, elenolic acid glucoside, decarboxymethyl elenolic acid, aldehydic form of decarboxymethyl elenolic acid, hydroxytyrosol acyclodihydroelenolate, phenylethyl primeveroside and comselpogoside) and flavonoids (gallocatechin, luteolin and apigenin). As can be seen in Figure 3, the rejection of flavonoids by the UP005 membrane was considerably high, above 80%, in comparison with that of the rest of the compounds. Therefore, the UP005 membrane achieved fractionation of polyphenols from wet olive pomace. Flavonoids were kept in the retentate (along with a high proportion of COD from the initial extract), whereas simple phenols, phenolic acids and secoridoids were recovered in the permeate. These molecules attract great interest nowadays because of their applications in nutraceutics, pharmacy and cosmetics [46,47]. Vanillic acid and decarboxymethyl elenolic acid were the less-rejected compounds, as shown in Figure 3. The obtention of a permeate enriched in these two compounds is of high interest due to the bioactive properties attributed to these molecules. Vanillic acid is a hydroxybenzoic acid that has shown interesting pharmacological effects, such as antiviral effects against the Epstein–Barr virus [48]. Furthermore, it has been effectively used to enhance the growth of microalgae, which can be later applied as a source of nutrients [49]. In the case of decarboxymethyl elenolic acid, this molecule has demonstrated antimicrobial effects [50,51], which suggests potential applications related to these antibiotic properties.

Then, the results derived from the LC-MS confirmed the satisfying performance of the UP005 membrane, because it allowed the purification of valuable phenolic compounds by retaining the concomitant organic matter.

### 3.3. Concentration of Phenolic Compounds by Means of Nanofiltration

#### 3.3.1. Permeate Flux in the Nanofiltration Step

**Figure 5:**
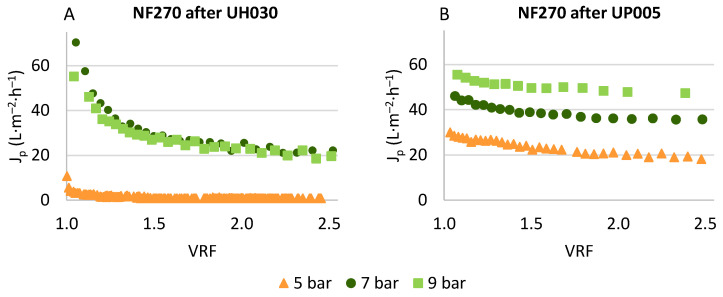
Permeate flux obtained with the NF270 membrane after the ultrafiltration of the extract with the UH030 membrane (**A**) and the UP005 membrane (**B**).

The permeate streams obtained with the UH030 and UP005 membranes were enriched in polyphenols with a potential application in industry. Therefore, the concentration of this extract was pursued by means of a nanofiltration process, employing the commercially available NF270 membrane. After its compaction, this membrane was characterized by testing its hydraulic permeability, obtaining 9 ± 1 L·h^−1^·m^−2^·bar^−1^. Subsequently, the ultrafiltration permeates were submitted to nanofiltration. Figure 5 shows the obtained permeate flux at each transmembrane pressure applied after the treatment of both streams.

Even though the results presented in both graphs of Figure 5 correspond to the same membrane (NF270), the evolution of permeate flux was very different in each case. This is explained by the different compositions of the feed solutions, which corresponded to the ultrafiltration permeate obtained with each ultrafiltration membrane. The objective of the ultrafiltration process was to purify the phenolic compounds (recovered in the permeate of the ultrafiltration) and reject as much organic matter as possible. As reflected in Figure 3, the rejection of COD achieved by the UH030 membrane was lower than the rejection obtained with the UP005 membrane. In consequence, the NF270 membrane suffered from higher fouling during the nanofiltration of the UH030 permeate (Figure 5A). An increment of the permeate flux can be observed in Figure 5A when the transmembrane pressure was increased from 5 to 7 bar. However, no increase in the permeate flux was observed with pressures higher than 7 bar. At 9 bar, a slight increment of the flux is shown in Figure 5A, but the evolution of the permeate flux was very similar to the one obtained at 7 bar, especially at high values of the VRF. For both pressures, stable values of permeate flux of 20 ± 0.2 L·h^−1^·m^−2^·bar^−1^ were obtained at a VRF of 2.5. As reported previously [19,29], higher pressures lead to higher deposition of solutes on the membrane surface, contributing to concentration polarization and fouling. The constant behavior of the permeate flux with the variation of transmembrane pressure is an indicator of the formation of a fouling layer on the membrane surface [52,53], which hindered solute diffusion throughout the membrane and reduced water transport too. When the pressure was increased up to 9 bar, this layer was compacted, contributing to flux reduction. This finding was also confirmed during the cleaning stage. The cleaning of the NF270 membrane after the processing of the UH030 permeate was more difficult than after treating the UP005 permeate. The detergent P3 Ultrasil (Ecolab, Spain) at 1% (*v*/*v*) had to be employed at 35 °C in order to recover the initial permeability of the NF270 membrane when the UH030 permeate was treated.

When the NF270 membrane was employed to treat the UP005 permeate, the permeate flux obtained increased linearly with transmembrane pressure, which indicated low fouling. This reduced fouling was in accordance with the high rejection of the organic matter that was observed during the ultrafiltration stage (Figure 3B), which led to a purified extract. In fact, the cleaning of the membrane after each experiment was performed simply by rinsing with tap water, and 100% of hydraulic permeability was recovered. This allowed the recycling of the membrane, which was reused during the whole process. The permeate flux obtained at all pressures was satisfactory. However, the membrane was more productive at 9 bar. At this pressure, a high permeate flux of 44.24 ± 0.08 L·h-1·m^−2^·bar^−1^ was achieved at a VRF of 2.5. Therefore, this pressure was selected at the most convenient for the concentration of the previously purified extract of wet olive pomace.

#### 3.3.2. Rejection Values in Nanofiltration

**Figure 6:**
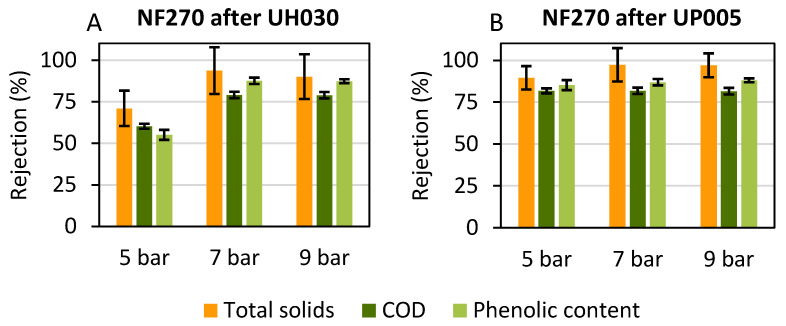
Rejection values obtained with the NF270 membrane at a volume reduction factor of 2.5 after the treatment of the permeate obtained with the UH030 membrane (**A**) and the UP005 membrane (**B**).

In order to evaluate the concentration of the aimed compounds, the rejection values obtained for total solids, COD and phenolic content were studied. These values are reflected in Figure 6.

The NF270 membrane was able to retain most of the solutes present in the permeate of the previous ultrafiltration. As already commented, the polyphenols present in the nanofiltration feed (that is the ultrafiltration permeate) were already highly purified. This means that the majority of the organic molecules present in this stream were phenolic compounds, especially in the stream corresponding to the permeate obtained with the UP005 membrane. For this reason, the rejection of total solids, COD and phenolic compounds was very similar, because the measurement of total solids and COD included the polyphenols.

At all studied pressures, the rejection values were high, in line with those from previous works [54,55]. During the treatment of the UH030 permeate, an increment in the rejection of total solids, COD and phenolic compounds could be observed when the pressure was increased from 5 bar to 7 bar (Figure 6A). In this case, this effect cannot be attributed to the compaction of the membrane with the pressure increment, because it was initially submitted to high pressure (see Section 2.3.2) to avoid further inconsistencies during the study. Instead, the increment in the rejection can be explained by an increase in the flux of water, which was not coupled to an increment in the flux of solutes. In an NF process, the diffusion of solutes plays an essential role in solutes transport [56]. However, this diffusion is not affected by TMP [57,58], whereas the flux of the solvent (water in this case) is highly dependent on the applied pressure. Then, increasing the pressure resulted in a high increment of the flux of water, whereas the flux of solutes did not suffer such a high rise. Figure 5A supports this reasoning, as a high increment in the permeate flux was observed when the pressure increased from 5 bar to 7 bar. In consequence, at 7 bar, the rejection of total solids, COD and phenolic compounds was higher than the values observed for 5 bar. At 9 bar, the rejection values were very similar to those at 7 bar. For instance, the rejection of total phenols was 87 ± 2% at 7 bar and 87 ± 1% at 9 bar. According to Figure 5, permeate flux did not increase either. As can be inferred from Figure 6A, this last increment in pressure did not derive any improvement for the membrane performance due to membrane fouling. On the contrary, the pressure increment from 5 bar to 7 bar enhanced the permeate flux and the rejection values, leading to a more efficient process, with a higher concentration of the desired compounds.

When the UP005 permeate was nanofiltered with the NF270 membrane, high rejections of COD, total solids and phenolic compounds were obtained at all pressures. High, satisfactory rejection values were achieved even when applying the lowest pressure. A slight increment in the rejection of the polyphenols of interest, COD and total solids can be observed in Figure 6B as TMP increased. Even though the selection of 5 bar to concentrate the purified extract of wet olive pomace could be suggested, high values of permeate flux and higher rejection of biophenols obtained at 9 bar should be taken into account too. Considering that the process was more productive at the highest pressure and that no fouling was observed, the application of 9 bar was selected for the concentration stage.

## 4. Conclusions

Ultrafiltration is a suitable membrane process to perform the purification of phenolic compounds from wet olive pomace. The UH030 membrane suffered from severe fouling that led to low values of permeate flux, whereas this fouling was not observed for the UP005 membrane. The UP005 membrane led to satisfactory results of permeate flux. Regarding rejections, this membrane rejected 70% of COD, allowing the passage of molecules of interest, such as several families of phenolic compounds of high added value. These could be later concentrated by a nanofiltration process by employing the NF270 membrane, which rejected more than 80% of the phenolic content at 9 bar and provided a high permeate flux. Then, the combination of ultrasound-assisted solid-liquid extraction with water (at 40 °C, for 45 min), ultrafiltration with the UP005 membrane (2.5 bar, 1.5 m·s^−1^) and nanofiltration with the NF270 membrane (9 bar) allowed the obtention of concentrated phenolic compounds at high purity.

## Figures and Tables

**Table 1:** Description of scientific contributions about the application of membrane technology to recover polyphenols from wet olive pomace.

Process	Working Mode	Permeate Flux (L·h^−1^·m^−2^)	Polyphenols Concentration	Reference
UF ^1^-NF ^2^-RO ^3^	Concentration	n.d. ^4^	200 mg GAE ^5^/L	[20]
UF-NF-RO	Concentration	n.d.	32.9 mg/L flavonoids	[21]
NF	Concentration	15 (20 bar)	1234.3 ± 54.0 mg GAE/L	[22]
UF-NF	Concentration	UF: 18 (2.5 bar); NF: 47 (9 bar)	882 mg TY ^6^/L	This work

^1^ Ultrafiltration. ^2^ Nanofiltration. ^3^ Reverse osmosis. ^4^ Not detailed. ^5^ Gallic acid equivalents. ^6^ Tyrosol equivalents.

**Table 2:** Characteristics of the employed membranes.

Parameter	UH030	UP005	NF270
MWCO (kDa) ^1^	30	5	0.3–0.4
Material	HPES ^2^	PES ^3^	Polyamide
Contact angle	56 ± 3° [27]	54.27 ± 3.48° [28]	15.9 ± 1.3° [29]
Manufacturer	Mycrodin Nadir	Mycrodin Nadir	DuPont
Process	UF	UF	NF

^1^ Molecular weight cut off. ^2^ Hydrophilic polyethersulfone. ^3^ Polyethersulfone.

**Table 3:** Characteristics of the aqueous extract of wet olive pomace.

Parameter	Concentration
COD ^1^ (mg/L)	8290 ± 548
Total solids (g/L)	9.05 ± 0.05
Total phenolic content (mg tyrosol/kg)	3970 ± 80
pH	5.4 ± 0.1
Conductivity (µS/cm)	1642 ± 18

^1^ Chemical oxygen demand.

## Data Availability

The raw data of this study are available on request.
